# Resveratrol Suppresses the Growth and Enhances Retinoic Acid Sensitivity of Anaplastic Thyroid Cancer Cells

**DOI:** 10.3390/ijms19041030

**Published:** 2018-03-29

**Authors:** Yi-Tian Li, Xiao-Ting Tian, Mo-Li Wu, Xu Zheng, Qing-You Kong, Xiao-Xin Cheng, Guang-Wen Zhu, Jia Liu, Hong Li

**Affiliations:** Liaoning Laboratory of Cancer Genetics and Epigenetics and Department of Cell Biology, College of Basic Medical Sciences, Dalian Medical University, Dalian 116044, China; liyitiannobless@sina.com (Y.-T.L.); txtdl2015@163.com (X.-T.T.); moliwusx@sina.com (M.-L.W.); dyzhengxu@yeah.net (X.Z.); kqydl@sina.com (Q.-Y.K.); xiaoxincheng@yahoo.com (X.-X.C.); jamesgwen@163.com (G.-W.Z.); jialiudl@aliyun.com (J.L.)

**Keywords:** resveratrol, anaplastic thyroid cancer, retinoic acid, drug resistance, CRABP2

## Abstract

Anaplastic thyroid cancer (ATC) is a highly lethal undifferentiated malignancy without reliable therapies. Retinoic acid (RA) has been employed to promote redifferentiation of thyroid cancers by increasing their I^131^ uptake and radio-sensitivity, but its effect(s) on ATCs has not yet been ascertained. Likewise, resveratrol induces cancer redifferentiation but, also in this case, its effects on ATCs remain unknown. These issues have been addresses in the current study using three human ATC cell lines (THJ-11T, THJ-16T, and THJ-21T) through multiple experimental approaches. The results reveal that RA exerts a small inhibitory effect on these cell lines. In comparison with normally cultured cells, the total cell number in resveratrol-treated THJ-16T and THJ-21T cultures significantly decreased (*p* < 0.05), and this effect was accompanied by reduced Cyclin D1 immuno-labeling, increased apoptotic fractions, and distinct caspase-3 activation. Resveratrol failed to inhibit growth but enhanced RA sensitivity of THJ-11T cells, suppressed peroxisome proliferator-activated receptor-β/δ (PPAR-β/δ), and upregulated cellular retinoic acid-binding protein 2 (CRABP2) and retinoic acid receptor beta (RAR-β) expression. Increased thyroglobulin (Tg) and E-cadherin levels and appearance of membranous E-cadherin were evidenced in resveratrol-treated THJ-11T cells. Our results demonstrate for the first time: (1) the therapeutic value of resveratrol by itself or in combination with RA in the management of ATCs, (2) the capacity of resveratrol to overcome RA resistance in ATC cells by reprogramming CRABP2/RAR- and fatty acid-binding protein 5 (FABP5)/PPAR-β/δ-mediated RA signaling, and (3) the redifferentiating potential of resveratrol in ATC cells.

## 1. Introduction

Thyroid cancer (TC) is the commonest endocrine malignancy and its incidence shows a steady increase in most countries [1]. In China, thyroid cancer is the fifth most frequent cancer for females with a rate of 16.32/100,000 [2]. According to the criteria of the World Health Organization (WHO), thyroid cancers are classified into five histological subtypes, including follicular carcinoma, papillary carcinoma, medullary carcinoma, undifferentiated anaplastic thyroid carcinoma (ATC), and others [3]. The majority of thyroid cancers are diagnosed as papillary carcinomas with promising prognosis [4], while ATC, though having a low incidence (1.7% among all thyroid cancer cases), accounts for 33–50% of TC-related death because of its aggressive growth, distal metastases formation and, especially, the lack of reliable adjuvant treatments [5]. Therefore, the efficient management of ATC patients becomes one of the main therapeutic challenges in the field.

Thyroidectomy is the first choice to treat ATCs, followed by chemoradiotherapy [6]. Docetaxel and cisplatin are the commonly used anti-ATC drugs but usually encounter drug resistance and cause severe adverse effects [7]. Retinoic acid (RA) is known as a differentiation inducer able to improve radioiodide uptake and radiosensitivity of thyroid cancers [8]. Although promising results were obtained in RA-treated ATC cells (FRO) [9], the clinical efficacy of RA-based redifferentiation therapy is still in dispute [10,11]. It is therefore necessary to explore alternative adjuvant agents for a better treatment of ATCs.

Resveratrol is a natural occurring product with multifaceted biological activities. A body of evidence demonstrates this polyphenol compound exerts inhibitory effects on many types of cancers including those with RA resistance [12] by inducing redifferentiation and apoptosis [13] and by inactivating cancer-associated signaling pathways [14,15,16]. More importantly, the anticancer dose of this polyphenol compound exerts minor cytotoxic effects on normal cells [15]. In this context, resveratrol may be a potential candidate for ATC therapy. However, the data concerning the effects of resveratrol on ATC cells and the comparative advantage of combining resveratrol with RA in ATC treatment remain limited. This study thus aims to address these issues using three human ATC cell lines.

## 2. Results

### 2.1. RA Resistance of Anaplastic Thyroid Cancer (ATC) Cell Lines

**Figure 1:**
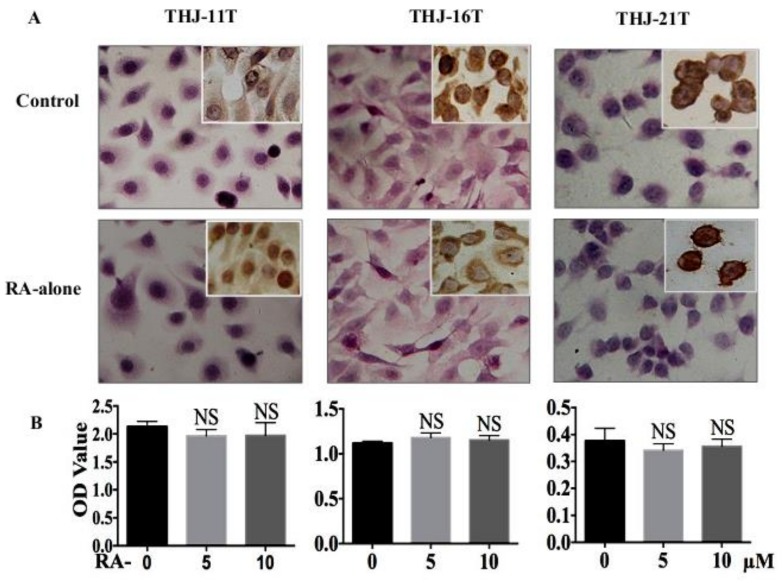

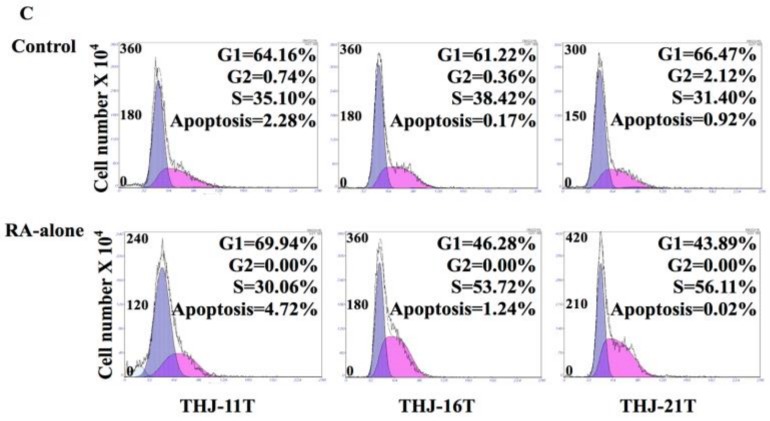
Lack of response of the three anaplastic thyroid cancer (ATC) cell lines to 10 µM retinoic acid (RA) treatment. (**A**) H/E staining (×40) and Cyclin D1 immunocytochemical staining (insets; ×40); (**B**) 3-[4,5-Dimethylthiazol-2-yl]-2,5-diphenyl-tetrazolium bromide (MTT) cell proliferation assay; (**C**) flow cytometry. Control, without resveratrol treatment; RA-alone, 10 µM retinoic acid treatment. NS, without statistical significance (*p* > 0.05); the error bars, the mean ± standard deviation; 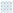, apoptosis peak; 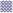, G1 phase; 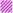, S phase; 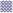, G2 phase.

The results of hematoxylin-eosin (H/E) morphological staining and immunocytochemical staining demonstrate that after 10 µM RA treatment for 48 h, no distinct cell death is observed in THJ-11T, THJ-16T, and THJ-21T cell populations (Figure 1A) and the levels and intracellular distribution patterns of Cyclin D1 remain unchanged (Insets of Figure 1A). 3-[4,5-Dimethylthiazol-2-yl]-2,5-diphenyl-tetrazolium bromide (MTT) cell proliferation assays (Figure 1B) reveal that after 5 µM and 10 µM RA treatment for 48 h, the optical density (OD) values of THJ-11T, THJ-16T, and THJ-21T cells are not significantly altered (*p* > 0.05) compared with that of the 0.2% dimethyl sulfoxide (DMSO)-treated counterparts (Control). Flow cytometry analysis (Figure 1C) shows no remarkable increase of the apoptotic fractions in the three ATC cell lines after 48 h RA treatment. S phase fractions of THJ-16T and THJ-21T are increased from 38.4% to 53.72% and from 31.3% to 56.11%, respectively, after 48 h 10 µM RA treatment. The cell cycle of RA-treated THJ-11T cells is similar to that of the untreated counterpart.

### 2.2. Resveratrol Suppresess the Growth of THJ-16T and THJ-21T Cells

**Figure 2:**
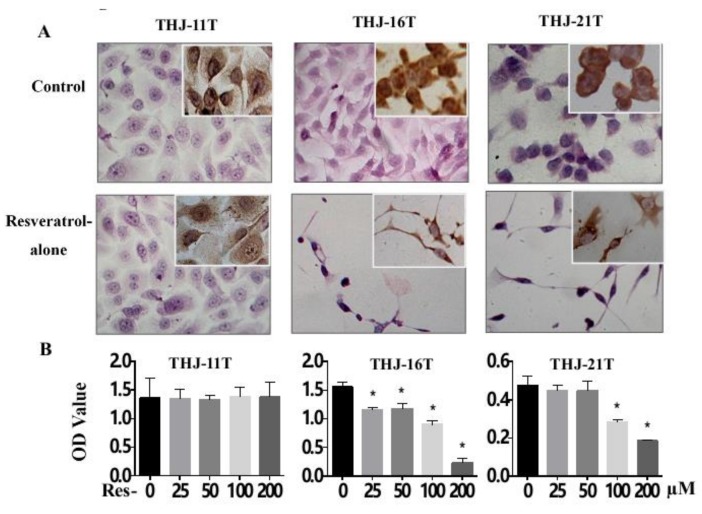

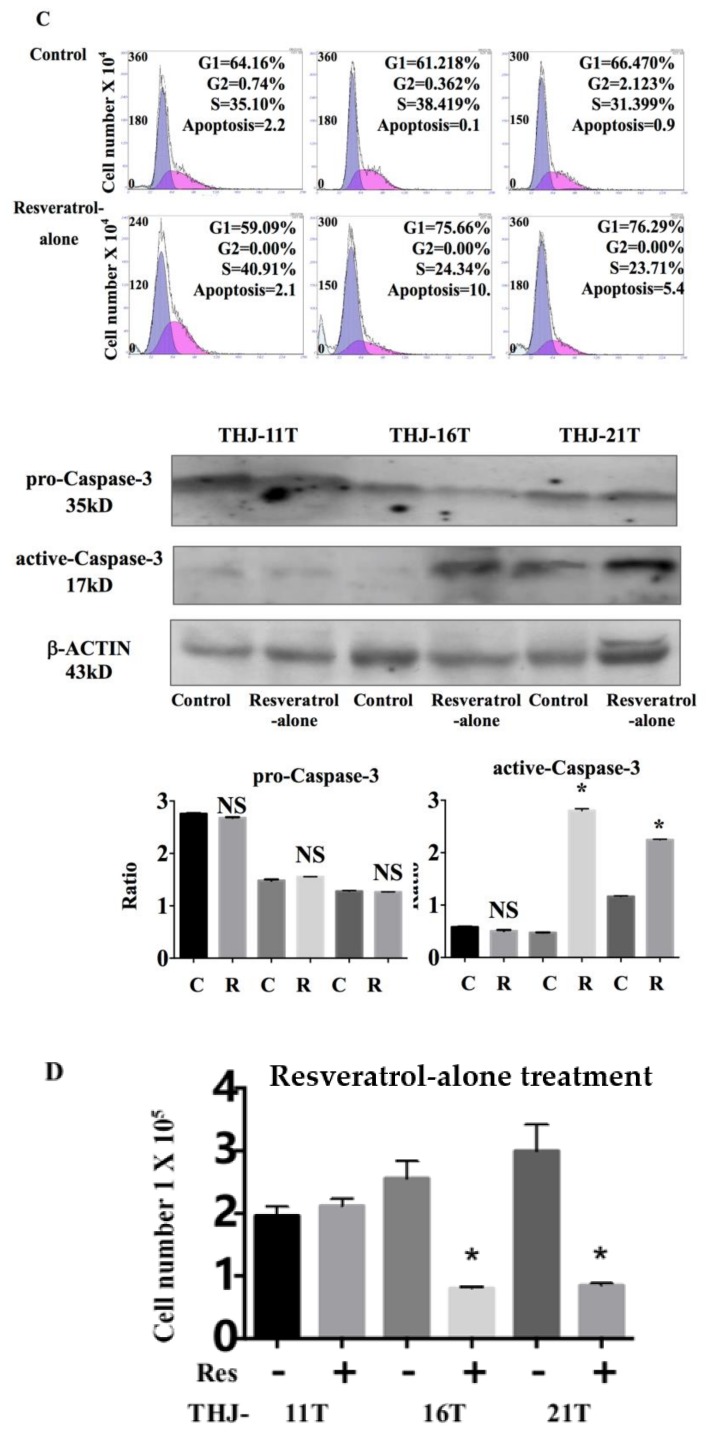
Different responses of the three ATC cell lines to resveratrol treatment. (**A**) H/E staining (×40) and Cyclin D1 immunocytochemical staining (insets; ×40) (**B**) MTT cell proliferation assay; (**C**) flow cytometry and Western blotting for pro-caspase-3 and active-caspase-3; (**D**) viable cell counting. *, with statistical significance (*p* < 0.05); the error bars, the mean ± standard deviation. Control, without resveratrol treatment; Res, 100 µM resveratrol treatment. NS, without statistical significance (*p* > 0.05); 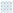, apoptosis peak; 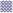, G1 phase; 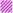, S phase; 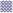, G2 phase.

H/E morphological staining demonstrates that after 100 µM resveratrol treatment for 48 h, THJ-16T and THJ-21T but not THJ-11T cells show extensive cell death (Figure 2A). MTT cell proliferation assay (Figure 2B) reveals that after 25 µM, 50 µM, 100 µM, and 200 µM resveratrol treatment for 48 h, the OD values of THJ-16T and THJ-21T cells decrease significantly in a dose-related fashion (*p* < 0.01) in comparison with those of the 0.2% DMSO (Control) and the resveratrol-treated THJ-11T cells. Flow cytometry analysis shows cell cycle arrest at G1 phase (76.3% and 75.7%) and increased apoptotic index (10.8% and 5.5%) of THJ-16T and THJ-21T, respectively, after 48 h 100 µM resveratrol treatment (Figure 2C). The total THJ-16T and THJ-21T cell numbers are significantly decreased (Figure 2D) to the extents of 68.6% and 71.9% after 48 h resveratrol treatment (*p* < 0.05). Meanwhile, remarkably reduced Cyclin D1 (Insets of Figure 2A) and 3.6-fold and 1.9-fold increase of the active form of caspase-3 (Figure 2C) are found in resveratrol-treated THJ-16T and THJ-21T, but not in THJ-11T cells.

### 2.3. Resveratrol Resistance of THJ-11T Cells

As shown in Figure 2D, resveratrol-treated THJ-11T cells show no distinct morphological change, and their total number displays a 7.4% increase in comparison with their normally cultured counterparts (*p* > 0.05). There is no significant difference of the OD values between 0.2% DMSO- and resveratrol-treated THJ-11T cells (*p* > 0.05). Flow cytometry analysis shows neither cell cycle arrest nor increased apoptotic index in 100 µM resveratrol-treated THJ-11T population. The patterns of Cyclin D1 immunocytochemical staining (insets of Figure 2A) and the states of pro- and active-caspase-3 (Figure 2C) show little changes in the resveratrol-treated population.

### 2.4. Resveratrol Reverses Retinoic Acid Resistance of THJ-11T Cells

**Figure 3:**
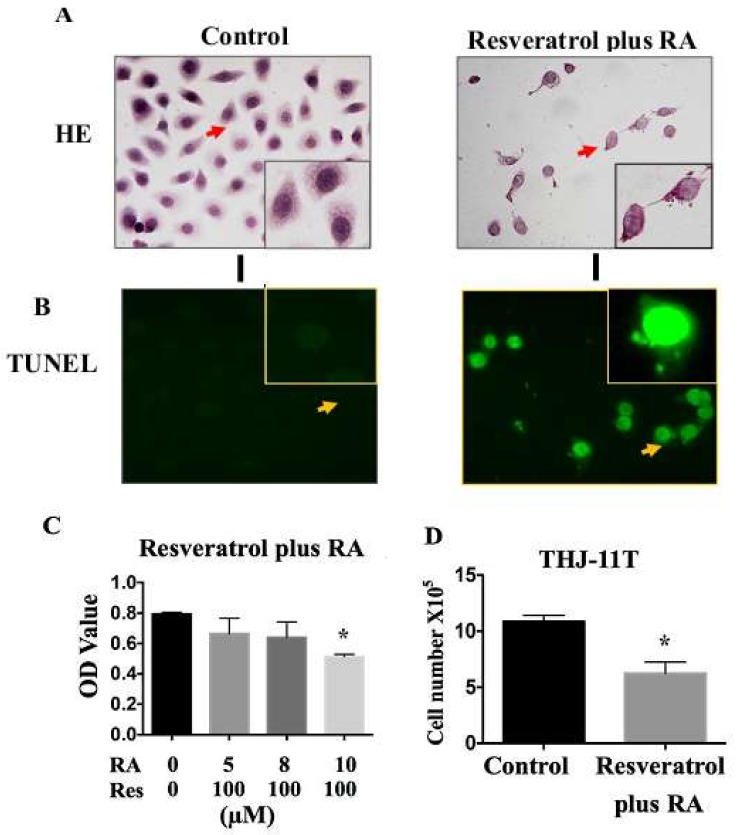

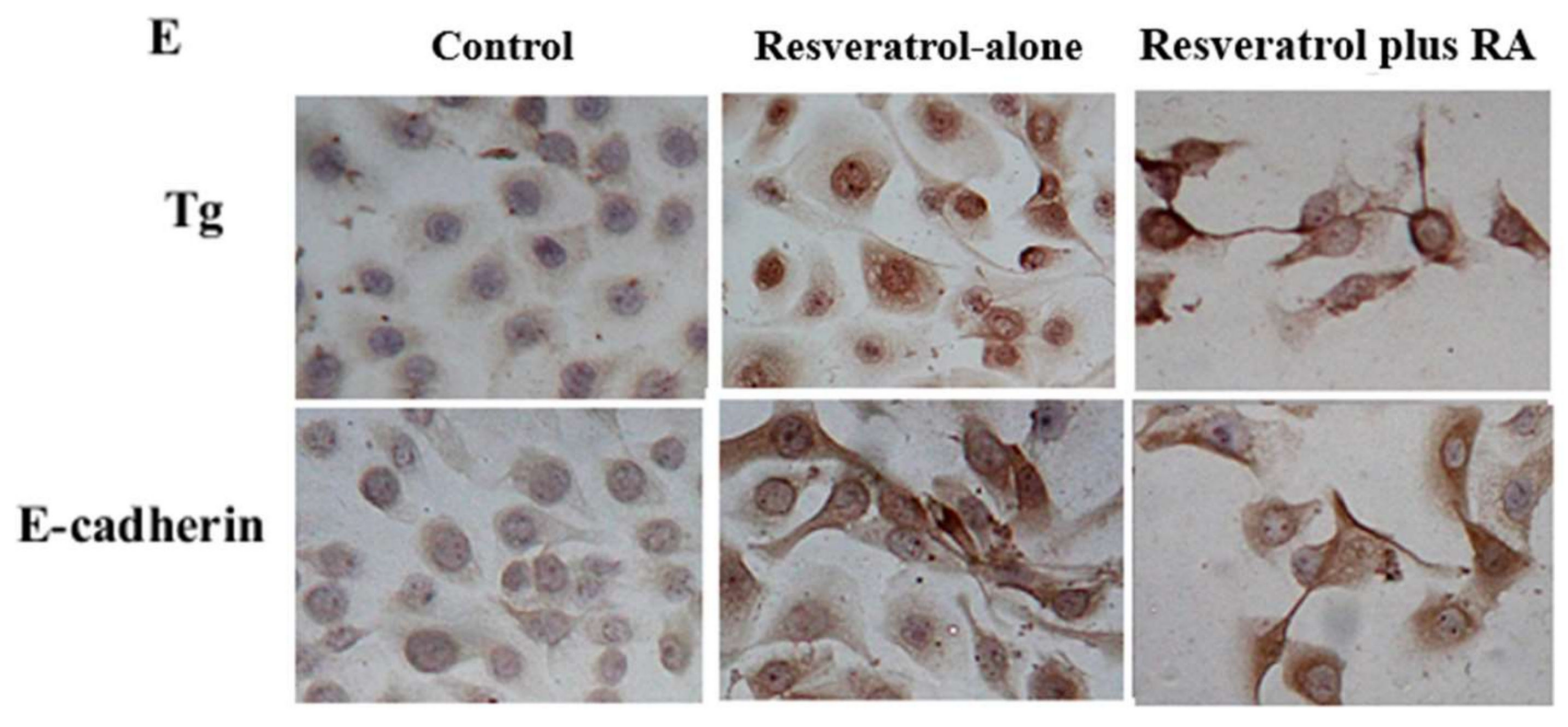
Resveratrol reverses the retinoic acid sensitivity of THJ-11T cells. (**A**) H/E staining (×40); (**B**) deoxynucleotidyl transferase-mediated dUTP-biotin nick and labeling assay (TUNEL) for apoptotic cell labeling (Green in color; ×40); (**C**) MTT cell proliferation assay; (**D**) viable cell counting; (**E**) immunocytochemical staining of thyroglobulin (Tg) and E-cadherin (×40). Control, cultured in 0.2% dimethylsulfoxide (DMSO)-containing medium; RA, retinoic acid treatment; Res, resveratrol treatment; Resveratrol plus RA, treated with a combination of 10 µM retinoic acid and 100 µM resveratrol for 48 h; Resveratrol-alone, 100 µM resveratrol; *, with statistical significance (*p* < 0.05); the error bars, the mean ± standard deviation. Arrows indicate the ports with higher magnification (×80) in the insets.

The combination of 100 µM resveratrol and 10 µM RA was employed to treat THJ-11T cells for 48 h. The results reveal a dose-related growth arrest in terms of decreased OD values (*p* < 0.05; Figure 3A and 3C), increased nonviable cell fraction (*p* < 0.05; Figure 3D), and frequent detection of deoxynucleotidyl transferase-mediated dUTP-biotin nick and labeling assay (TUNEL)-positive cells (Figure 3B) in comparison with cells cultured in 0.2% DMSO-containing medium. Immunocytochemical staining (Figure 3E) reveals that thyroglobulin (Tg) expression is extremely low in THJ-11T cells and remarkably increased after resveratrol treatment. E-cadherin is expressed at low levels and distributed in the cytoplasm; however, after resveratrol treatment, it becomes upregulated and appears at THJ-11T plasma membrane. Similar findings are evidenced in THJ-11T cells treated with the resveratrol and RA combination.

### 2.5. Significant Upregulation of Cellular Retinoic Acid-Binding Protein 2 (CRABP2) in Resveratrol-Treated THJ-11T Cells

**Figure 4:**
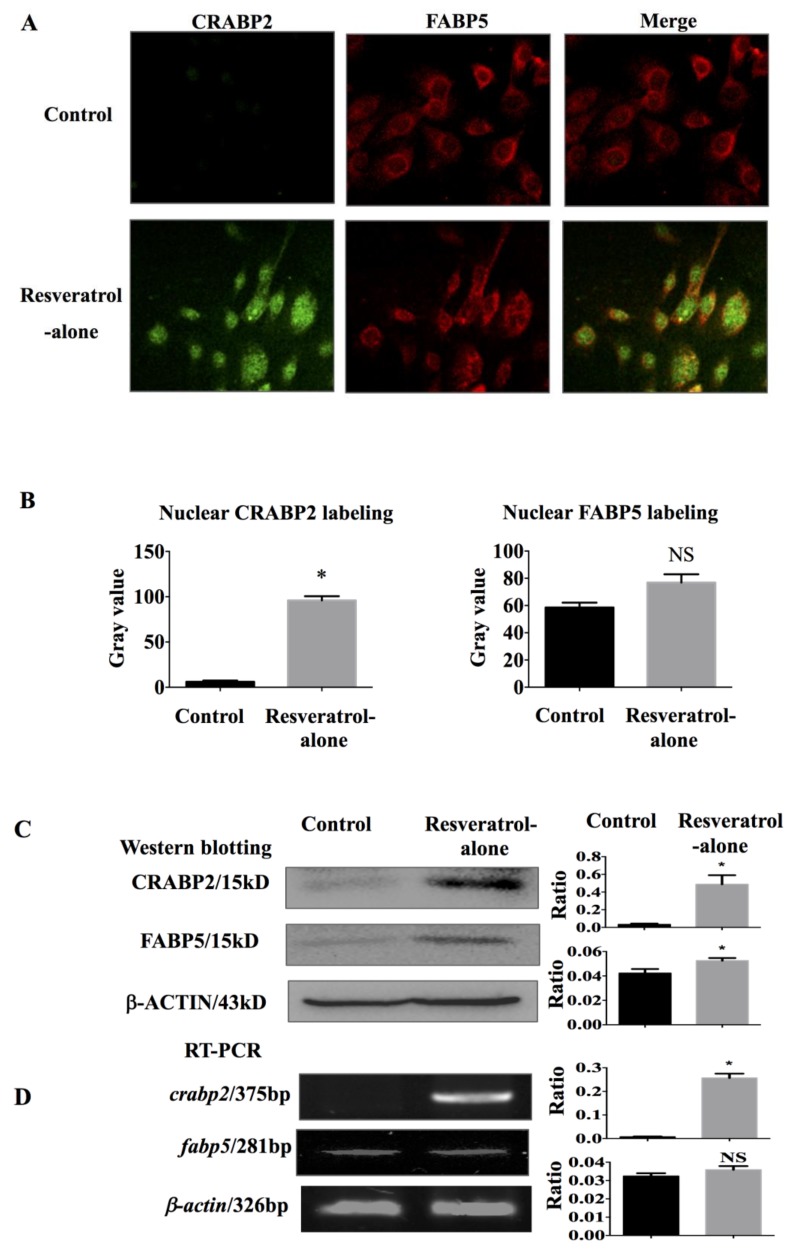
Differential expression of CRABP2 and FARP5 in THJ-11T cells in the absence or presence of 100 µM resveratrol. (**A**) Double immunofluorescent labeling (×40); (**B**) ImageJ (Version 1.0, National Institutes of Health, Bethesda, MD, USA)-based quantification of nuclear labeling of CRABP2 and FABP5; (**C**) Western blotting; (**D**) RT-PCR. Control, 0.2% DMSO-treated cells; Resveratrol-alone, 100 µM resveratrol treatment; Ratio, ratio between the levels of the target molecules and that of β-ACTIN/*β-actin*; *, with statistical significance (*p* < 0.05); NS, no statistical significance (*p* > 0.05); the error bars, the mean ± standard deviation.

Because cellular retinoic acid-binding protein 2 (CRABP2) and fatty acid-binding protein (FABP5) are factors that determine the response of cancer cells to RA [17], their expression patterns in THJ-11T cells without and with resveratrol treatment were examined by CRABP2- and FABP5-oriented double immunofluorescent labeling. As shown in Figure 4A, FABP5 is stably expressed in THJ-11T cells irrespective of resveratrol treatment; CRABP2 labeling is extremely weak in normally cultured cells and becomes distinct especially in the nuclei cells treated for 48 h with 100 µM resveratrol. The mean gray value of nuclear CRABP2 labeling in resveratrol-treated cells is 15.8 folds higher than that in control cells; no statistical difference of nuclear FABP5 gray values between THJ-11T cells treated or not with resveratrol treatment are found (*p* > 0.05; Figure 4B). The gray density analyses of the Western blotting results demonstrate a 16-fold increase of CRABP2 and a 24.3% increase of FABP5 in 100 µM resveratrol-treated cells (Figure 4C). In accordance, RT-PCR reveals that *crabp2* transcript is almost undetectable in normally cultured THJ-11T cells but is 34-fold upregulated in 100 µM resveratrol-treated cells (Figure 4D). The level of *fabp5* expression is slightly (10.4%) increased after 48 h resveratrol treatment.

### 2.6. Differential Response of Retinoic Acid Receptor (RAR)-β and Peroxisome Proliferator-Activated Receptor (PPAR)-β/δ to Resveratrol

**Figure 5:**
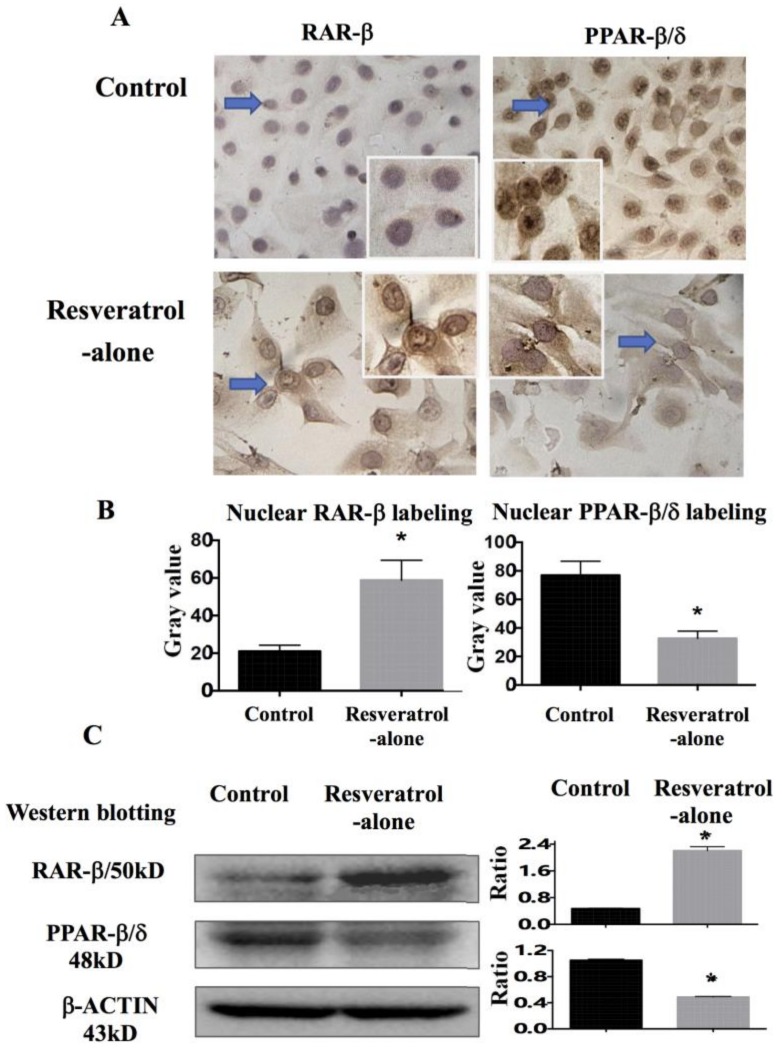
Immunocytochemical and Western blotting demonstration of resveratrol-induced alterations of RAR-β and PPAR-β/δ expression and their intracellular distribution patterns in THJ-11T cells. (**A**) Immunocytochemical staining (×40); (**B**) ImageJ-based quantification of nuclear labeling of RAR-β and PPAR-β/δ; (**C**) Levels of RAR-β and PPAR-β/δ determined by Western blotting. Control, 0.2% DMSO-treated cells; Resveratrol, 48 h 100 µM resveratrol treatment. Arrows indicate the portions in higher magnification in the insets (×80). Ratio, ratio between the levels of the target molecules and that of β-ACTIN; *, with statistical significance (*p* < 0.05); NS, no statistical significance (*p* > 0.05); the error bars, the mean ± standard deviation. Gray values, nuclear RAR-β and PPAR-β/δ immunocytochemical staining densities.

It has been known that CRABP2 and FABP5 signals are closely related to retinoic acid receptor (RAR)-β and peroxisome proliferator-activated receptor (PPAR)-β/δ, respectively [17]. Therefore, the protein levels of RAR-β and PPAR-β/δ in THJ-11T cells in the absence or presence of resveratrol treatment were examined by immunocytochemical staining and Western blotting. It was found that RAR-β is weakly stained in the cytosol and that PPAR-β/δ is strongly labeled in the nuclei of THJ-11T cells; increased RAR-β with distinct nuclear translocation and reduction of cytosolic and nuclear PPAR-β/δ labeling are observed in cells treated with 100 µM resveratrol for 48 h (Figure 5A). The mean gray value of nuclear RAR-β labeling increases 2.8 folds and that of nuclear PPAR-β/δ shows a 57% reduction in resveratrol-treated cells (Figure 5B). The results of Western blotting demonstrate a 5-fold increase of RAR-β and a 56% reduction of PPAR-β/δ in resveratrol-treated THJ-11T cells (Figure 5C).

## 3. Discussion

Undifferentiated thyroid carcinoma or anaplastic thyroid cancer (ATC), though characterized by a low incidence, is the most lethal thyroid malignancy [18] because the majority of ATC patients die within one year after diagnosis [19]. In addition to the highly aggressive behavior of this type of cancer, the lack of efficient adjuvant therapy is the main reason for the extremely poor prognosis of ATCs [18]. RA has been commonly used in combination with radiotherapy to treat aggressive thyroid cancers and it can decrease the viability of FRO ATC cell line through anti-proliferative and cytotoxic activities [9]. To elucidate whether these effects also occur in other ATC cells, three ATC cell lines (THJ-11T, -16T, and -21T) are employed and treated with RA in this study. The results clearly demonstrate that RA exerts a small inhibitory effect on these cell lines in terms of proliferation activity, cyclin D1 labeling pattern, and cell death compared with their untreated counterparts. These findings thus suggest the frequent establishment of RA resistance in ATCs and the necessity to explore more reliable agents against this highly aggressive malignancy. Resveratrol is a potential candidate because of its nontoxic properties in normal cells and its ability to induce redifferentiation and apoptosis in cancer cells [20] and to inhibit cancer-associated signaling [21,22,23]. So far, no data are available concerning the effects of resveratrol on ATC cells.

It has been found that resveratrol effectively suppresses the growth and induces neuronal differentiation of RA-resistant medulloblastoma cells [12]. We therefore considered that this non-toxic polyphenol compound might exert similar effects on the three RA-resistant ATC cell lines. Our results reveal distinct inhibitory effects of 100 µM resveratrol on THJ-16T and THJ-21T cells in the forms of remarkable growth arrest (68.6% and 71.9%), G1-phase accumulation with downregulated Cyclin D1 expression, and increased apoptotic fractions (10.3% and 5.5%) with distinct casepase-3 activation. Additionally, these findings prove, for the first time, the therapeutic value of resveratrol in the treatment of ATCs, especially those with RA resistance. Nevertheless, resveratrol is not a universal anti-ATC agent because THJ-11T cells show a limited response to resveratrol under the same experimental conditions, suggesting the necessity to conduct a resveratrol-based anti-ATC therapy in a personalized manner. In this context, THJ-11T and other two resveratrol-sensitive ATC cell lines would be of value in exploring the specific molecular elements that determine resveratrol therapeutic efficacy. Meanwhile, it is necessary to find alternative approaches to treat ATC cells that are sensitive neither to RA nor to resveratrol.

A body of evidence reveals that resveratrol can sensitize cancer cells to conventional anticancer drugs. For instance, it enhances the effects of temozolomide on glioblastoma-initiating cells by promoting redifferentiation [24] and of cisplatin on non-small-cell lung cancer cells by inducing mitochondrial dysfunction and cell apoptosis [25]. We therefore presumed that resveratrol might be helpful in reversing RA resistance in ATC cells. This speculation was tested by treating THJ-11T cells with a resveratrol and RA combination (Res/RA). Differently from the situation found in THJ-11T cells treated with 100 µM resveratrol or 10 µM RA only, decreased proliferation activity accompanied with increased fractions of nonviable cells and TUNEL-positive labeling cells were evidenced in Res/RA-treated populations. Our results thus confirm the efficacy of resveratrol to reverse RA resistance in ATC cells and suggest the potential clinical utility of this combination in the management of ATCs that are sensitive neither to resveratrol nor to RA. This notion is further supported by the redifferentiation tendency of resveratrol-treated THJ-11T in terms of increased Tg and E-cadherin production and, especially, the appearance of membranous E-cadherin localization that, as shown in the current study, are extremely low and even absent in undifferentiated and, especially, anaplastic thyroid cancers [26,27,28,29]. As the next step, the potential of resveratrol to improve I^131^ uptake of ATC cells should be investigated.

CRABP2 has been known as the central player of RA tumor suppression, and, in contrast, FABP5 has been known as an RA tumor promotion signaling; these molecules function by delivering RA to its corresponding nuclear receptors, i.e., RAR-β and PPAR-β/δ, respectively [30,31,32]. It is, therefore, proposed that the ratio of CRABP2 and FABP5 and the status of expression of their corresponding nuclear receptors may determine RA sensitivities of cancer cells [17,33,34]. Given the evidence that resveratrol can enhance RA sensitivity, the expression patterns of CRABP2, RAR-β, FABP5, and PPAR-β/δ in THJ-11T cells and the impact of resveratrol in them were analyzed. The results clearly demonstrate that the ratio of CRABP2/FABP5 is imbalanced THJ-11T cells because of an extremely low CRABP2 expression; this situation is reversed upon resveratrol treatment with a remarkably increased level of CRABP2 and largely unchanged FABP5 expression. Concurrently, a distinct RAR-β nuclear accumulation and a reduced PPAR-β/δ expression and nuclear labeling are found in resveratrol-treated cells. On the basis of the above findings, it would be reasonable to consider that CRABP2/RAR-β- and FABP5/PPAR-β/δ-mediated RA signaling pathways are reprogrammed by resveratrol, which may enhance RA sensitivity of THJ-11T cells and, presumably, of RA-resistant ATC cases. In this context, resveratrol would be of potential value in improving the efficacy and in avoiding the adverse effects of RA in the treatment of ATCs.

## 4. Materials and Methods

### 4.1. Thyroid Cancer Cell Lines and Culture

THJ-11T, 16T, and 21T cell lines [35] were kindly provided by Quentin Liu, Institute of Cancer Stem Cell, Dalian Medical University. They were cultured in 1640 medium with l-glutamine (Hyclone, Logan, UT, USA), supplemented with 10% fetal bovine serum (Gibco, Grand island, NY, USA) for THJ-11T and THJ-21T, and with 5% fetal bovine serum for THJ-16T. An amount of 5 × 10^4^/mL cells were plated onto culture dishes (Nunc A/S, Roskilde, Denmark) at 37 °C and 5% CO_2_ for 24 h before the experiments were performed. For haematoxylin and eosin (H/E), immunocytochemical staining and flow cytometry analyses (Becton Dickinson, San Jose, CA, USA), dozens of cell-bearing coverslips were prepared under the same experimental conditions using coverslip-preparation dishes (Jet Biofile Tech. Inc., Guangzhou, China; China invention patent No. ZL200610047607.8) for multiple experimental purposes.

### 4.2. Reagents and Cell Treatments

Resveratrol and all-trans retinoic acid (RA; Sigma-Aldrich, St. Louis, MO, USA) were dissolved in dimethylsulfoxide (DMSO; Sigma-Aldrich) and diluted with culture medium to 100 µM and 10 µM, respectively, as the working concentrations, just before use [36]. The cells were treated with 100 µM resveratrol or 10 µM RA for 48 h and observed at 8 h intervals. Normally cultured cells and cells treated with 0.2% DMSO were used as the untreated, background, and insensitive controls. Cell numbers and viability were checked at 12 h intervals, and the cell-bearing coverslips were fixed in cold acetone for morphological and immunocytochemical staining or in 4% paraformaldehyde (pH 7.4) for TUNEL apoptosis assay and immunofluorescence (IF) assay. The experimental groups were designed in triplicate, and each of the experiments was repeated at least three times. The data obtained were summarized and statistically quantified for a confidential conclusion.

### 4.3. Evaluation of Cell Proliferation and Death

To elucidate the cellular response of ATC cells to resveratrol treatment, H/E staining, viable/nonviable cell counting (Automated Cell Counter, Bio-Rad, Singapore), and 3-[4,5-Dimethylthiazol-2-yl]-2,5-diphenyl-tetrazolium bromide (MTT) cell proliferation assay were performed on coverslips bearing THJ-11T, THJ-16T, and THJ-21T cells treated or not with the drugs by the methods described elsewhere [37]. Flow cytometry (Becton Dickinson, San Jose, CA, USA) was employed to determine the cell cycle phases and apoptotic incidence, and the data obtained were analyzed with a with MOD FIT software (Version 5.0, Verity Software House Inc., Topsham, ME, USA) [37]. Cells treated with 0.2% DMSO were used as controls. The DNA fragmentation assay in THJ-11T cells on coverslips, treated with both RA and resveratrol, was performed by using a modification of the terminal deoxynucleotide transferase (TdT)-mediated dUTP-biotin nick end-labeling method (TUNEL; Roche Inc., Basel, Switzerland). Cells on coverslips without drug treatment were used as a negative control. Fluorescence microscopy (BX51, Olympus, Tokyo, Japan) was used to observe and photograph the cells on coverslips.

### 4.4. Protein Preparation and Western Blotting

Western blotting was conducted using antibodies against CRABP2 (Proteintech, Chicago, IL, USA; 1:200), FABP5 (Proteintech, Chicago, IL, USA; 1:200), RAR-β (Bioss. Inc., Beijing, China; 1:150), PPAR-β/δ (Bioss. Inc., Beijing, China; 1:200), pro-caspases-3, and active-caspases-3 (Abcam Inc., Cambridge, UK; 1:500 and 1:500). The experiment was performed by the method described elsewhere [15]. Briefly, the sample proteins (20 µg/well) were separated by 10% SDS-PAGE electrophoresis and transferred to polyvinylidene difluoride membrane (Amersham, Buckin ghamshire, UK). The membrane was blocked in 5% skimmed milk (Sigma-Aldrich) Tris-buffered saline (TBS-T) (10 mM Tris–HCl, pH 8.0, 0.5% Tween 20 and 150 mM NaCl) at 4 °C overnight. After three washes with TBS-T, the membrane was incubated for 3 h with the primary antibody at room temperature, followed by 1 h incubation with horseradish peroxidase (HRP)-conjugated anti-rabbit IgG (Zymed Lab Inc., San Francisco, CA, USA). The enhanced chemiluminescence system (Roche, Penzberg, Germany) was used to detect the bound antibody. The labeling signal was removed with a stripping buffer (62.5 mM Tris–HCl, pH 6.7, 100 mM 2-mercaptoethanol, 2% sodium dodecyl sulfate (SDS), and the membrane was reprobed with another antibody until all the parameters were examined.

### 4.5. Immunocytochemical Staining and Double Immunofluorescent Labeling

Immunocytochemical staining was conducted on cell-bearing coverslips by the method described elsewhere [15]. The antibodies used were: Cyclin D1 (Bioss Inc., Beijing, China; 1:100), Tg (Bioss. Inc., Beijing, China; 1:200), E-cadherin (Bioss. Inc., Beijing, China; 1:100), RAR-β (Bioss. Inc., Beijing, China; 1:150), and PPAR-β/δ (Bioss. Inc., Beijing, China; 1:200). Cells incubated in 0.2% DMSO-containing medium were used as a control. The color reaction was performed by using 3,3′-diaminobenzidine tetrahydrochloride (DAB) after the binding of the primary antibody (Vector Laboratories, Burlingame, CA, USA). For double immunofluorescent staining (IF), mouse anti-CRABP2 and rabbit anti-FABP5 were employed in the working concentrations of 1:120. Briefly, the cell-bearing coverslips were washed with phosphate-buffered solution (PBS, pH 7.4), incubated in 3% H_2_O_2_ for 10 min and then with anti-CRABP2 (1:120; Proteintech, Chicago, IL, USA) and anti-FABP5 (1:120; Proteintech, Chicago, IL, USA) at 4 °C for one night in a humid chamber. Finally, the coverslips were co-incubated with FITC-conjugated goat anti-mouse IgG and PE-conjugated goat anti-rabbit IgG (both 1:100; Santa Cruz Biotechnology, Santa Cruz, CA, USA) at 37 °C for 60 min in the dark, sealed with fluorescence mounting medium, and observed and imaged under a fluorescence microscope (BX53F, Olympus, Tokyo, Japan).

### 4.6. RNA Isolation and RT-PCR

The RNA was isolated from THJ-11T cell lines treated or not with resveratrol for 48 h and subjected to reverse transcription (RT) [37]. For RT, 0.5 µg of each RNA sample was added to 20 µL of RT reaction mixture (Takara, Inc., Ltd., Dalian, China) containing 4 µL of MgCl_2_, 2 µL of 10 RNA PCR buffer, 9.5 µL of RNase-free distilled H_2_O, 2 µL of dNTP mixture, 0.5 µL of RNase inhibitor, 1 µL of AMV reverse transcriptase, and 1 µL of oligo dT-adaptor primer. The reaction was carried out at 55.8 °C for 30 min, at 98 °C for 5 min, and at 58 °C for 5 min [38]. Polymerase chain reaction (PCR) was conducted with a pair of primers specific for *crabp2* (forward primer: 5′-ATGCCCAACTTCTCTGGCAA-3′; reverse primer: 5′-CGTCATGGTCAGGATCAGTT-3′), *fabp5* (forward primer: 5′-AGCAGCTGGAAGGAAGATGG-3′; reverse primer: 5′-CTGATGCTGAACCAATGCAC-3′), and *β-actin* (forward primer: 5′-GCATGGAGTCCTGTGGCAT-3′, reverse primer: 5′-CTAGAAGCATTTGCGGTGG-3′). The PCR for *crabp2* was performed as follows: after initial denaturation for 2 min at 85 °C and 2 min at 94 °C, the samples were subjected to 30 cycles at 94 °C for 30 s, 59 °C for 30 s, 72 °C for 60 s. Then, after a final extension time of 10 min at 72 °C, the samples were stored at 4 °C. The PCR for *fabp5* was performed as follows: after initial denaturation for 5 min at 95 °C, the samples were subjected to 40 cycles at 94 °C for 45 s, 55 °C for 45 s, 72 °C for 90 s. Then, after a final extension time of 10 min at 72 °C, they were stored at 4 °C [39]. Agarose gels (1.2%) containing ethidium bromide (0.5 µg/mL) were prepared for the separation of the PCR products, and UVP Biospectrum Imaging System (UVP, Inc, Upland, CA, USA) was used to visualize and photograph the samples.

### 4.7. ImageJ-based Quantification of Nuclear Translocation

As described described elsewhere [40], five view fields in each immunocytochemically stained cell coverslip were photographed under a microscope (×40), and the images were adjusted by RGB (red green blue)/HSB (hue saturation brightness) stack until only the green (CRABP2), red (FABP5), or brown (RAR-β or PPAR-β/δ) labeling were shown. The nuclei were selected using ROI (regions of interest) Manager tool, and their gray values were evaluated and summarized by calculating the mean ± standard deviation (SD). One-way ANOVA was employed to evaluate the statistical difference of the gray values between controls and resveratrol-treated cells.

### 4.8. RA Sensitivity Assay of Resveratrol-Treated THJ-11T Cells

THJ-11T cells were treated with 100 µM resveratrol, 10 µM RA, or their combination for 48 h. To elucidate the response of resveratrol-treated THJ-11T cells to RA, H/E staining, viable/nonviable fractionation, TUNEL labeling, and MTT cell proliferation assay were performed in THJ-11T cells treated differently by methods described elsewhere [41,42].

### 4.9. Statistical Analyses

Each experiment was conducted for three times, and the data obtained were analyzed together. The results of the MTT cell proliferation assay and cell counting were evaluated with ANOVA and the independent-samples *t*-test. The bar graphs present the mean ± standard deviation (SD) of separate experiments (*n* ≥ 8). When required, *p*-values are provided in the figures and their legends.

## 5. Conclusions

The effectiveness of resveratrol and retinoic acid against anaplastic thyroid cancer (ATC) cells is investigated in this study. The results show that all three ATC cell lines employed are insensitive to RA treatment, while two of them are sensitive to resveratrol in terms of growth arrest, G1 phase accumulation, and extensive apoptosis. Resveratrol exerts little inhibitory effect on THJ-11T ATC cells but upregulates Tg and E-cadherin expression and efficiently reverses their RA resistance by activating CRABP2/RAR-mediated tumor suppression signaling. Our findings thus demonstrate, for the first time, the therapeutic advantages of resveratrol in suppressing ATC cell growth by itself or in combination with RA. We suggest that this nontoxic polyphenol compound is of potential value in improving the clinical management of the lethal ATCs, especially those resistant to RA.
